# Loss of KDM5B ameliorates pathological cardiac fibrosis and dysfunction by epigenetically enhancing ATF3 expression

**DOI:** 10.1038/s12276-022-00904-y

**Published:** 2022-12-08

**Authors:** Bo Wang, Yong Tan, Yunkai Zhang, Sheng Zhang, Xuewen Duan, Yuyu Jiang, Tong Li, Qingqing Zhou, Xingguang Liu, Zhenzhen Zhan

**Affiliations:** 1 Key Laboratory of Arrhythmias of the Ministry of Education of China, Research Center for Translational Medicine, Shanghai East Hospital, Tongji University School of Medicine, 200120 Shanghai, China; 2 Department of Pathogen Biology, Naval Medical University, 200433 Shanghai, China; 3 Shanghai Fourth People’s Hospital, Tongji University School of Medicine, 200081 Shanghai, China; 4 Department of Liver Surgery, Shanghai Institute of Transplantation, Renji Hospital, Shanghai Jiao Tong University School of Medicine, 200127 Shanghai, China

**Keywords:** Cardiomyopathies, Experimental models of disease, Epigenetics, Histone analysis

## Abstract

Excessive cardiac fibrosis is central to adverse cardiac remodeling and dysfunction leading to heart failure in many cardiac diseases. Histone methylation plays a crucial role in various pathophysiological events. However, the role of histone methylation modification enzymes in pathological cardiac fibrosis needs to be fully elucidated. Here, we identified lysine demethylase 5B (KDM5B), a histone H3K4me2/me3 demethylase, as a key epigenetic mediator of pathological cardiac fibrosis. KDM5B expression was upregulated in cardiac fibroblasts and myocardial tissues in response to pathological stress. KDM5B deficiency markedly ameliorated cardiac fibrosis, improved cardiac function, and prevented adverse cardiac remodeling following myocardial infarction (MI) or pressure overload. KDM5B knockout or inhibitor treatment constrained the transition of cardiac fibroblasts to profibrogenic myofibroblasts and suppressed fibrotic responses. KDM5B deficiency also facilitated the transformation of cardiac fibroblasts to endothelial-like cells and promoted angiogenesis in response to myocardial injury. Mechanistically, KDM5B bound to the promoter of activating transcription factor 3 (*Atf3*), an antifibrotic regulator of cardiac fibrosis, and inhibited ATF3 expression by demethylating the activated H3K4me2/3 modification, leading to the enhanced activation of TGF-β signaling and excessive expression of profibrotic genes. Our study indicates that KDM5B drives pathological cardiac fibrosis and represents a candidate target for intervention in cardiac dysfunction and heart failure.

## Heart disease: Enzyme that promotes fibrosis identified

An enzyme that is increased in heart tissues during cardiac fibrosis could provide a valuable treatment target. Injury or excessive pressure in the heart during disease can trigger cardiac remodeling: changes in the heart’s shape, size and function that often result in heart failure. A key feature of this remodeling is cardiac fibrosis, where heart cells are overactivated triggering excessive collagen deposition. Targeted therapies are limited. A team led by Zhenzhen Zhan at Tongji University and Xingguang Liu at Naval Medical University in Shanghai, China, identified an enzyme, lysine demethylase 5B (KDM5B), involved in promoting cardiac fibrosis. In mice models, KDM5B expression was increased in cardiac fibroblasts, whereas KDM5B deficiency improved cardiac fibrosis and restored heart function. Further examination showed that KDM5B bound to the promoter of an anti-fibrotic regulator, inhibiting its expression and promoting fibrosis.

## Introduction

Ischemic and hypertrophic heart diseases, which are the two most prominent cardiovascular diseases that lead to heart failure, share the common pathological hallmark of adverse cardiac remodeling. Cardiac fibrosis is the pivotal characteristic manifestation of adverse cardiac remodeling induced by cardiac insults, which can be elicited by ischemia, hemodynamic overload, neurohumoral activation and cytokines^1^. Cardiac fibrosis is typically characterized by the overactivation and excessive proliferation of myofibroblasts, which disrupts the balance between the synthesis and degradation of extracellular matrix proteins^2^. While appropriate fibrosis is beneficial in maintaining cardiac structure to prevent cardiac rupture in some conditions, such as myocardial infarction (MI), the overactivated fibrotic response causes excessive deposition of collagen fibers in the localized or entire myocardium to alter myocardial architecture and decrease myocardial compliance, resulting in the development of cardiac dysfunction, arrhythmias, and heart failure^3,4^. A large amount of literature demonstrates that pathological cardiac fibrosis represents a principal pathway mediating clinical outcomes in heart failure for patients who are exposed to ischemic and nonischemic heart stress events^5^. However, an effective therapeutic approach for cardiac fibrosis is very limited, which urges us to further explore the key mechanisms underlying pathological cardiac fibrosis.

Among the multiple types of cells in the heart, cardiac resident fibroblasts are considered to be the most predominant sources of myofibroblasts, accounting for approximately 11% of the total cells in the adult mouse heart^6,7^. Under normal physiological conditions, fibroblasts are in a quiescent state. However, in the injured heart, fibroblasts transdifferentiate into activated myofibroblasts and significantly contribute to cardiac remodeling in response to multiple pathological stresses^8^. Apart from mediating cardiac fibrosis, fibroblasts can also remodel the impaired heart under certain circumstances. For instance, previous studies have shown that fibroblasts can adopt an endothelial phenotype and promote angiogenesis to repair the injured heart^9,10^. Therefore, the precise regulation of fibroblast fate under stress conditions is vital for improving cardiac function and preventing pathological cardiac fibrosis. However, the mechanisms controlling fibroblast fate and maintaining their appropriate activation state remain incompletely understood.

Multiple regulatory mechanisms are involved in modulating cardiac fibrosis^11^. Epigenetic mechanisms, including histone methylation, play essential roles in regulating chromatin structure and gene expression^12^. It has been shown that alterations in the histone methylation profiles of various cardiac cell types can be induced by different pathological stimuli^13^. These alterations can contribute to the imbalance in homeostasis in multiple cell types in the heart, including increasing the expression of fibrosis-associated genes in fibroblasts^13^. A previous study showed that histone lysine methyltransferase DOT1L (disruptor of telomeric silencing 1-like) contributed to the profibrotic response in cardiac fibroblasts by enhancing the activation of transforming growth factor β (TGF-β)/SMAD signaling^14^, suggesting that manipulating the histone methylation profile has great potential in overcoming pathological cardiac fibrosis. However, whether other histone methylation modification enzymes play key roles in controlling cell fate and profibrotic responses in cardiac fibroblasts needs to be further explored.

Lysine-specific demethylase 5B (KDM5B), which is also known as PLU1 or Jumonji AT-rich interactive domain 1B (JARID1B), mediates the demethylation of transcription-activated trimethylation and dimethylation of H3 at lysine 4 (H3K4me3 and H3K4me2), suppressing the expression of target genes^15^. Some studies have demonstrated that abnormal expression of KDM5B is associated with the development of multiple disorders, including the promotion of tumorigenesis and inflammatory diseases^16,17^. However, the role of KDM5B in cardiovascular diseases is still unknown. Here, we found that the loss of KDM5B significantly blocked the activation of cardiac fibroblasts and promoted angiogenesis by increasing the methylation level of H3K4me2/3 and promoting the expression of activating transcription factor 3 (ATF3) in cardiac fibroblasts, which repressed pathological cardiac fibrosis and prevented cardiac dysfunction. Our study elucidated the critical epigenetic regulatory role of KDM5B in the pathogenesis of aberrant cardiac fibrosis.

## Materials and methods

### Reagents

Recombinant mouse TGF-β protein (7666-MB) was purchased from R&D Systems (Minneapolis, MN). Angiotensin II (AngII) (CSN10313) was purchased from CSNpharm (Chicago, IL). Antibodies against α-smooth muscle actin (α-SMA) (ab5694, for immunoblot analysis), phospho-SMAD3 (SMAD family member 3) (ab52093), and KDM5B (ab181089, for immunofluorescence analysis) were obtained from Abcam (Cambridge, UK). Antibodies against collagen type I (AB765p) were obtained from Millipore (Billerica, MA). Antibodies against α-SMA (5228, for immunofluorescence and immunohistochemistry) were obtained from Sigma‒Aldrich (Darmstadt, Germany). Antibodies against collagen type III (NB600-594) were obtained from Novus Biologicals (Littleton, CO). Antibodies against phospho-SMAD2 (SMAD family member 2) (3108), SMAD2 (5339), SMAD3 (9523), phospho-ERK (extracellular signal-regulated MAP kinase) (9106), ERK (4696), phospho-JNK (c-Jun N-terminal kinase) (4668), JNK (9252), phospho-p38 (4511), p38 (9212), phospho-p65 (3033 S), p65 (8242), Glyceraldehyde-3-phosphate dehydrogenase (GAPDH) (5174) and ATF3 (18665) were obtained from Cell Signaling Technology (Danvers, MA). Antibodies against KDM5B (A301-813A, for immunoblot analysis) were obtained from Bethyl Laboratories (Montgomery, TX).

### Mice

Wild-type C57BL/6 mice were obtained from Sipper BK Laboratory Animals (Shanghai, China). KDM5B-knockout (KO) mice were generated by using the CRISPR/Cas9 system. The 428 bases containing the JmjC domain, which is responsible for its demethylase activity, were deleted from the *Kdm5b* gene, resulting in a frameshift in the *Kdm5b* gene and the loss of function of KDM5B. All mice were maintained under specific pathogen-free level barrier conditions in the Experimental Animal Center of Tongji University, and all experiments were performed in accordance with procedures approved by the National Institutes of Health Guide for the Care and Use of Laboratory Animals.

### Animal models

For AngII-induced pressure overload, an osmotic minipump (Alzet Model 2004; Alza Corp) containing a 28-day infusion of saline or AngII (1000 ng/kg/min) was implanted subcutaneously in the mice. For the MI model, left anterior descending coronary artery permanent ligation was performed as previously described^18^. Briefly, after anesthetization by inhalation of isoflurane (2.5 L/min isoflurane at 1.5 L/min of O_2_), 8–10-week-old male mice were ventilated with a small animal ventilator at a tidal volume of 0.45 ml for assisted breathing. The chest cavity was opened through a left thoracotomy to expose the heart, and the left anterior descending branch was permanently ligated with an 8–0 silk ligature. The chest and skin were closed using 5–0 and 4–0 ligatures, respectively. After removing the tracheal tube, the mice were positioned on a warming pad at 37 °C until full consciousness was restored. At the end of the experimental schedule following AngII infusion or the MI surgery, all mice were euthanized by cervical dislocation after anesthetization with an intraperitoneal overdose of sodium pentobarbital (70 mg/kg body weight), followed by dissection of heart tissue and relevant experimental investigations. Two-dimensional echocardiography (Vevo 2100; VisualSonics) with an M-mode and 30-MHz frequency probe was used to measure the cardiac function of the anesthetized mice at prescheduled time points in a blinded manner.

### Cell culture and RNA interference

Primary cardiac fibroblasts were isolated from the left ventricular myocardium of mice. Briefly, the dissected hearts from 2-week-old mice were minced into small pieces of approximately 1 mm^3^ size with scissors and then incubated with a prewarmed digestion buffer containing 0.125% trypsin at 37 °C for 10 min. The supernatants were collected after repeated mixing with a pipette, and the remaining tissues were processed 4–5 times until digestion was complete. Cardiac fibroblast fractions were collected by centrifugation and resuspended in DMEM with 10% fetal bovine serum (Gibco, MA). The resuspended cells were plated on treated tissue culture dishes (Corning, NY) in a humidified incubator. Two predesigned *Kdm5b*-specific siRNAs or control siRNA targeting different sequences from different companies (Dharmacon, CO; Santa Cruz Biotechnology, TX) were used for *Kdm5b* silencing. Adherent cardiac fibroblasts were transfected with *Kdm5b* siRNA or control siRNA utilizing Lipofectamine RNAiMAX (Thermo Fisher Scientific, MA) according to the manufacturer’s protocol.

### RNA isolation and quantitative PCR (Q-PCR)

Total RNA was extracted from myocardial tissues or cultured cardiac fibroblasts with TRIzol reagent (Invitrogen, CA) and reverse-transcribed with a First Strand cDNA Synthesis Kit (TOYOBO, Shanghai, China) according to the manufacturer’s protocol. The cDNA of each sample was used to assess changes in the expression of different genes by using QuantStudio 6 (Applied Biosystems, Thermo Fisher Scientific). The data were normalized to *Gapdh* expression.

### Transcriptome microarray analysis

Microarray analysis was performed with Agilent Whole Mouse Genome 4 × 44 K arrays. Total RNA was extracted from the samples using TAKARA RNAiso (TAKARA, Dalian, China) according to the manufacturer’s standard operating procedures. The total RNA was quality checked by Agilent Bioanalyzer 2100 (Agilent Technologies, CA) electrophoresis and purified using an RNeasy mini kit (QIAGEN, Dusseldorf, Germany) and RNase-Free DNase Set (QIAGEN). Labeling, hybridization and washing were performed according to the Agilent guidelines. The completed hybridized microarrays were scanned using an Agilent Microarray Scanner (Agilent Technologies, CA). Microarray signals and background information were retrieved using Feature Extraction Software 10.7 (Agilent Technologies) and normalized using GeneSpring Software 12.6.1 (Agilent Technologies). The cutoffs for differential expression were set as an absolute fold-change >2 and a corrected *p* value <0.05. Transcriptome microarray data are available at the Gene Expression Omnibus under the accession number GSE197223.

### RNA sequencing (RNA-seq)

Cardiac fibroblasts were isolated from the left ventricular myocardium of KDM5B-knockout or littermate WT mice on Day 7 after MI. RNA samples were isolated from cardiac fibroblasts for library construction and sequenced with an Illumina NovaSeq 6000 to generate 150 bp paired-end reads. The reads were mapped to the reference mouse genome (mm10) using HISAT2 with default parameters. The fragment per kilobase of transcript per million mapped reads (FPKM) values were used to evaluate differential gene expression. The clustering of differentially expressed genes, Gene Ontology (GO) functional enrichment analysis and other data analyses were performed using custom programs. The RNA sequencing data are available at the Gene Expression Omnibus under the accession number GSE213746.

### Tube formation analysis

Forty-eight-well plates were coated with Growth Factor Reduced Corning Matrigel Matrix (Corning Life Sciences, MA) and polymerized for 30 min at 37 °C according to the manufacturer’s protocol. Equal amounts of cultured cardiac fibroblasts isolated from KDM5B-KO and littermate WT mice were trypsinized and seeded into the coated plates. After 4–6 h of incubation, tube formation mediated by cardiac fibroblasts was photographed.

### Immunoblot analysis

Total proteins were extracted from myocardial tissues or cultured cardiac fibroblasts with cell lysis buffer (Cell Signaling Technology) containing a protease inhibitor cocktail (Merck, Darmstadt, Germany) and phenylmethylsulfonyl fluoride. The concentrations of the extracted proteins were measured with the Pierce BCA protein assay kit (Thermo Fisher Scientific). Equal amounts of proteins were used to perform immunoblot analysis.

### Histological analysis

Isolated hearts were prepared for histological analysis using the paraffin sectioning method as previously described^19^. Briefly, the harvested hearts were fixed in 4% paraformaldehyde and then dehydrated, embedded in paraffin, and cut into a series of 5-μm-thick sections. Masson’s trichrome staining was performed to evaluate the fibrotic scar area by using a trichrome staining kit according to the manufacturer’s instructions. Immunofluorescent and immunohistochemical staining was performed as previously described^10^. Digital images were captured using a Leica microscope system (Wetzlar, Germany).

### Chromatin immunoprecipitation (ChIP)

Cardiac fibroblasts were cross-linked using 1% (vol/vol) formaldehyde, and the reaction was terminated with 0.125 M glycine. Then, protein‒DNA complexes in the harvested crosslinked cells were sonicated in ice water to form random fragments between 200 bp and 500 bp in size. The supernatant extracts were collected by centrifugation and then subjected to ChIP analysis according to the protocol of the Chromatin Immunoprecipitation Kit (Millipore). Target gene promoter sequences in input DNA and the recovered DNA immunocomplexes were detected by Q-PCR. The data were normalized to the corresponding DNA input control. The primers used to detect the mouse *Atf3* gene promoter were as follows: 5’-TGACAGGGGCCATTTGAG AAATC-3’ (forward) and 5’-CCTCTGAGCAACTCCACAGAGCA-3’ (reverse).

### Statistical analysis

The statistical significance of differences between two groups was determined by unpaired Student’s t test. For comparisons of more than two groups, one- or two-way analysis of variance (ANOVA) with the least significant difference t test was performed. The data were analyzed with Prism 7 (GraphPad), and *p* values < 0.05 were considered to be statistically significant.

## Results

### KDM5B expression is upregulated in cardiac fibroblasts after myocardial injury

**Fig. 1:**
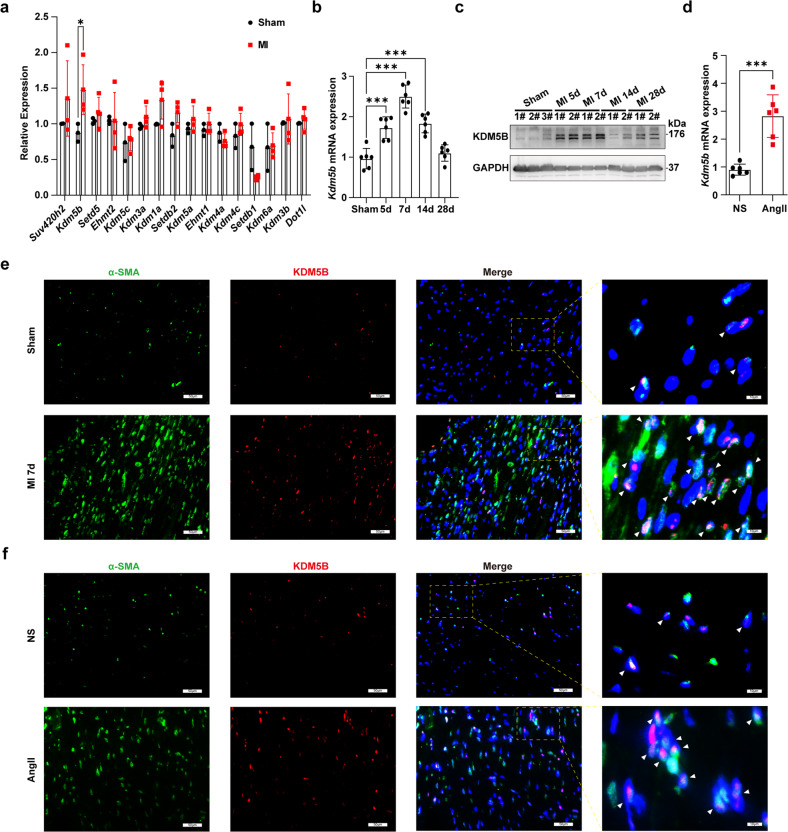
KDM5B expression is increased in cardiac fibroblasts after myocardial injury. **a** Q-PCR analysis of the mRNA expression levels of methylation modification enzymes in myocardial tissues from WT mice on Day 7 after MI or sham operation (*n* = 3 for sham group, *n* = 4 for MI group). **b**, **c** Q-PCR analysis of *Kdm5b* mRNA (*n* = 6 mice per group) or immunoblot analysis of KDM5B protein expression in myocardial tissues from WT mice on the indicated day after MI or sham operation. # indicates the serial numbers of the mice. **d** Q-PCR analysis of *Kdm5b* mRNA expression in myocardial tissues from WT mice on Day 28 following AngII or normal saline (NS) infusion (*n* = 6 mice per group). **e** Representative immunofluorescence staining of KDM5B (red) or α-SMA (green) in myocardial tissues from WT mice on Day 7 after MI or sham operation. Scale bar, 50 μm (magnified: 10 μm). **f** Representative immunofluorescence staining of KDM5B (red) or α-SMA (green) in myocardial tissues from WT mice on Day 28 after AngII or NS infusion. Scale bar, 50 μm (magnified: 10 μm). *p < 0.05, ***p < 0.001. Unpaired Student’s *t* test (**a**, **d**) or one-way ANOVA (**b**) was performed.

To screen histone methylation modification enzymes that modulate the progression of cardiac fibrosis after myocardial injury, we first measured changes in their expression in myocardial tissues after MI or pressure overload. Among these histone methylation modification enzymes, the increase in *Kdm5b* mRNA expression was the most significant in the ischemic myocardium of mice after MI (Fig. 1a). KDM5B expression in the myocardium was upregulated in a time-dependent manner, peaking on Day 7 after MI (Fig. 1b, c). We further observed changes in KDM5B expression in response to pressure overload and found that *Kdm5b* expression was markedly elevated in the myocardium after AngII infusion-induced pressure overload (Fig. 1d). We then determined the cellular type that contributed to the increase in KDM5B expression in the myocardium after pathological stress. Immunofluorescence analysis showed that the increase in KDM5B expression was predominantly distributed in α-SMA-positive cardiac fibroblasts but not cardiomyocytes in myocardial tissues subjected to MI or AngII infusion (Fig. 1e, f and Supplementary Fig. 1a–d). Furthermore, TGF-β treatment increased the mRNA and protein expression levels of KDM5B in primary cardiac fibroblasts isolated from the mouse myocardium (Supplementary Fig. 1e, f). These results suggest that the histone demethylase KDM5B, which is increased in cardiac fibroblasts, is involved in pathological processes in response to different types of myocardial injury.

### KDM5B deficiency ameliorates cardiac fibrosis and adverse cardiac remodeling after MI

**Fig. 2:**
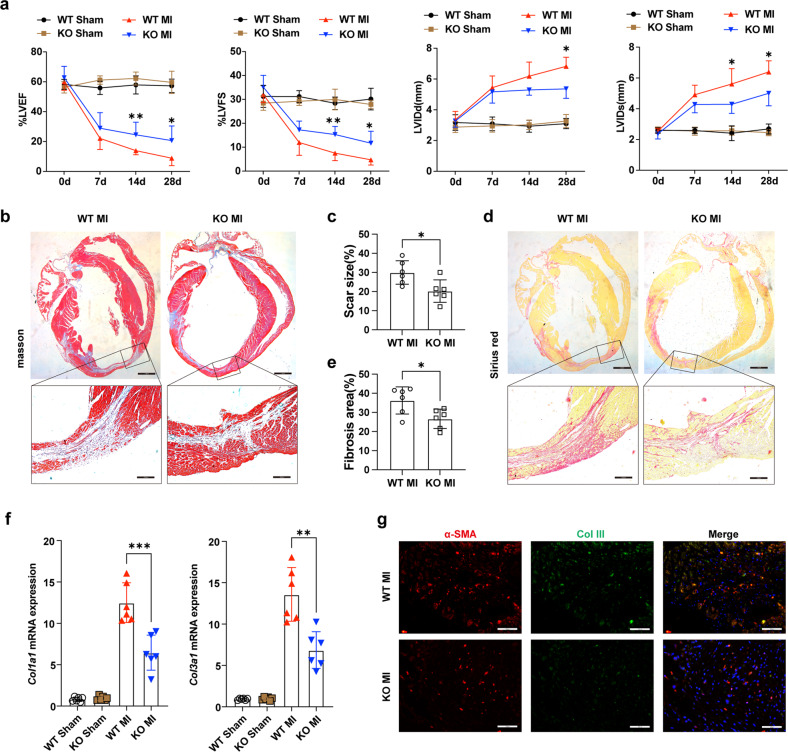
KDM5B deficiency protects the heart from dysfunction and adverse cardiac remodeling after MI. **a** Echocardiographic measurement of the LVEF, LVFS, left ventricular end-diastolic internal dimension (LVIDd), and left ventricular end-systolic internal dimension (LVIDs) of KDM5B-KO or WT mice at baseline (Day 0) and on the indicated day after MI or sham operation (*n* = 6 mice per group). **b**–**e** Representative Masson’s trichrome staining images and quantitation of the scar size (**b**, **c**) or Sirius red staining images and quantitation of the fibrosis area (**d**, **e**) in myocardial tissues from KDM5B-KO or WT mice on Day 28 after MI. *n* = 6 mice per group. Scale bar, 1.6 mm (upper), 200 μm (bottom). **f** Q-PCR analysis of *Col1a1* and *Col3a1* mRNA levels in myocardial tissues from KDM5B-KO or WT mice on Day 14 after MI or sham operation (*n* = 6 mice per group). **g** Representative immunofluorescence staining of α-SMA (red) and Col III (green) in myocardial tissues from KDM5B-KO or WT mice on Day 14 after MI (*n* = 6 mice per group). Scale bar, 50 μm. *p < 0.05, **p < 0.01, ***p < 0.001. Unpaired Student’s *t* test (**c**, **e**) or ANOVA (**a**, **f**) was performed.

KDM5B-deficient mice on a C57BL/6 background were generated with the CRISPR/Cas9 system, which mediated a 428 bp deletion in the Jmjc domain that is responsible for its demethylase activity and the accompanying frameshift mutation in the *Kdm5b* gene (Supplementary Fig. 2a). There was almost no mRNA or protein expression of KDM5B in cardiac fibroblasts from KDM5B-KO mice (Supplementary Fig. 2b, c). In addition, immunofluorescent staining showed no expression of KDM5B in the nuclei of KDM5B-deficient cardiac fibroblasts (Supplementary Fig. 2d). Echocardiography analysis indicated no difference in cardiac function between KDM5B-KO and control littermate WT mice under physiological conditions (Supplementary Fig. 2e, f). Masson’s trichrome staining suggested that KDM5B-deficient mice did not show any adverse cardiac remodeling compared with WT mice under physiological conditions (Supplementary Fig. 2g). We next explored the role of KDM5B in cardiac function and remodeling after MI. KDM5B-KO mice showed increased percentages of left ventricular ejection fraction (LVEF) and left ventricular fractional shortening (LVFS) compared with control littermate WT mice on Days 14–28 after MI (Fig. 2a). Reduced left ventricular dimensions and left ventricular volumes were also observed in KDM5B-KO mice after MI (Fig. 2a and Supplementary Fig. 3a), indicating that KDM5B deficiency improved contractile function and reduced left ventricular dilation of the heart after MI. KDM5B deficiency decreased the heart weight-to-body weight (HW/BW) and heart weight-to-tibia length (HW/TL) ratios (Supplementary Fig. 3b, c). Masson’s trichrome and Sirius red staining revealed markedly decreased scar and fibrosis areas in the myocardium of KDM5B-KO mice compared with WT mice after MI (Fig. 2b–e). The mRNA expression levels of fibrosis-related genes, including collagen type I alpha 1 chain (*Col1a1*), collagen type III alpha 1 chain (*Col3a1*), fibronectin 1 (*Fn1*), cellular communication network factor 2 (*Ccn2*), *Tgfb*, and actin alpha 2 smooth muscle (*Acta2*), in the myocardium were dramatically suppressed by KDM5B deficiency (Fig. 2f and Supplementary Fig. 3d). Immunofluorescence and immunohistochemical staining indicated a limited number of α-SMA-positive myofibroblasts in the myocardium and reduced expression of collagen III in the myofibroblasts of KDM5B-KO mice (Fig. 2g and Supplementary Fig. 3e, f). These data indicate that KDM5B exacerbates cardiac fibrosis mediated by overactivated myofibroblasts, leading to worse cardiac dysfunction and adverse cardiac remodeling after MI.

### KDM5B deficiency attenuates cardiac dysfunction and fibrosis following pressure overload

**Fig. 3:**
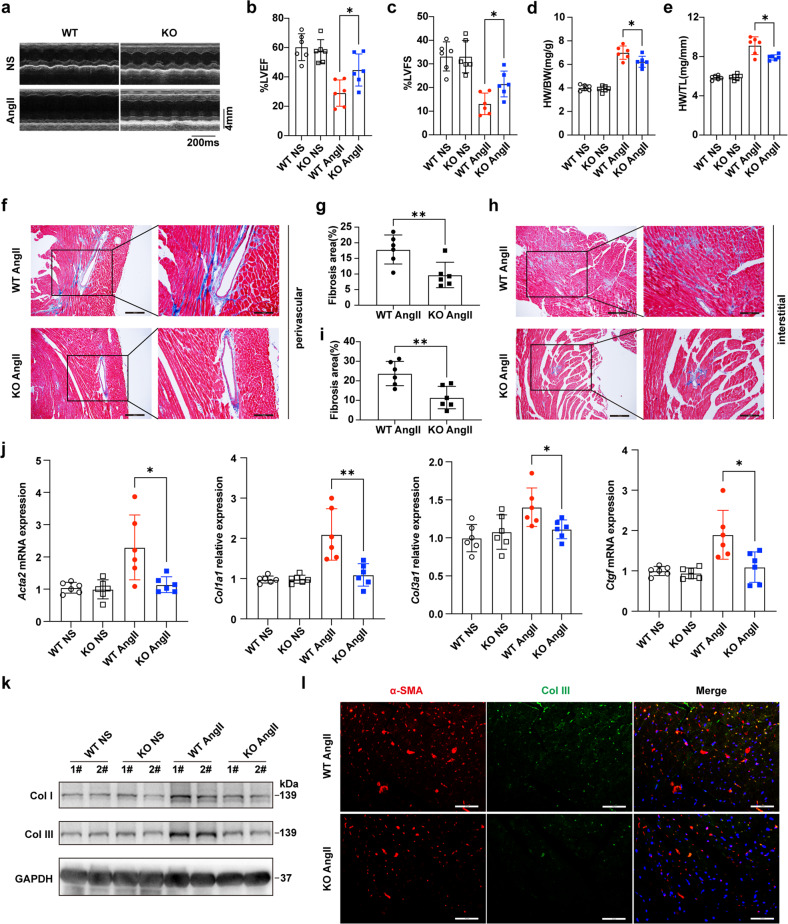
KDM5B deficiency prevents pressure overload-induced cardiac dysfunction and cardiac fibrosis. **a** Representative echocardiographic M-mode images of left ventricles from KDM5B-KO or littermate control WT mice on Day 28 after AngII or normal saline (NS) infusion. **b**, **c** Echocardiographic measurement of the LVEF (**b**) and LVFS (**c**) of KDM5B-KO or WT mice on Day 28 after AngII or NS infusion (*n* = 6 mice per group). **d**, **e** The ratio of heart weight to body weight (HW/BW) (**d**) and the ratio of heart weight to tibia length (HW/TL) (**e**) of KDM5B-KO or WT mice on Day 28 after AngII or NS infusion (*n* = 6 mice per group). **f**–**i** Representative Masson’s trichrome images and quantitation of perivascular (**f**, **g**) or interstitial (**h**, **i**) fibrosis in myocardial tissues from KDM5B-KO or WT mice on Day 28 after AngII or NS infusion (*n* = 6 mice per group). Scale bar, 200 μm (left), 100 μm (right). **j** Q-PCR analysis of *Acta2*, *Col1a1*, *Col3a1* and *Ctgf* mRNA expression levels in myocardial tissues from KDM5B-KO or WT mice on Day 28 after AngII or NS infusion (*n* = 6 mice per group). **k** Immunoblot analysis of Col I and Col III protein expression in myocardial tissues from KDM5B-KO or WT mice on Day 28 after AngII or NS infusion. **l** Representative immunofluorescence staining of α-SMA (red) and Col III (green) in myocardial tissues from KDM5B-KO or WT mice on Day 28 after AngII infusion. Scale bar, 50 μm. *p < 0.05, **p < 0.01. Unpaired Student’s *t* test (**g**, **i**) or one-way ANOVA (**b**–**e**, **j**) was performed.

We further investigated the role of KDM5B in cardiac function and fibrosis after pressure overload. KDM5B deficiency significantly improved cardiac function after AngII infusion-induced pressure overload (Fig. 3a–c). KDM5B-deficient mice showed decreased heart weight to body weight and tibia length ratios (Fig. 3d, e). Masson’s trichrome staining showed that myocardial perivascular and interstitial fibrosis was dramatically attenuated in KDM5B-deficient mice compared to WT mice subjected to AngII infusion (Fig. 3f–i). The mRNA levels of fibrosis-related genes, including *Acta2*, *Col1a1*, *Col3a1*, and connective tissue growth factor (*Ctgf*), were markedly decreased in the myocardial tissues of KDM5B-deficient mice following AngII infusion (Fig. 3j). KDM5B deficiency inhibited the protein expression of collagen I and collagen III in myocardial tissues after AngII infusion (Fig. 3k). Immunofluorescence staining showed decreased collagen III production in the limited α-SMA-positive myofibroblasts in the myocardium of KDM5B-deficient mice after AngII infusion (Fig. 3l and Supplementary Fig. 3g). These results demonstrate that KDM5B deficiency protects against cardiac dysfunction, cardiac fibrosis and pathological remodeling triggered by pressure overload.

### The loss of KDM5B suppresses fibrotic responses and the transition of cardiac fibroblasts

**Fig. 4:**
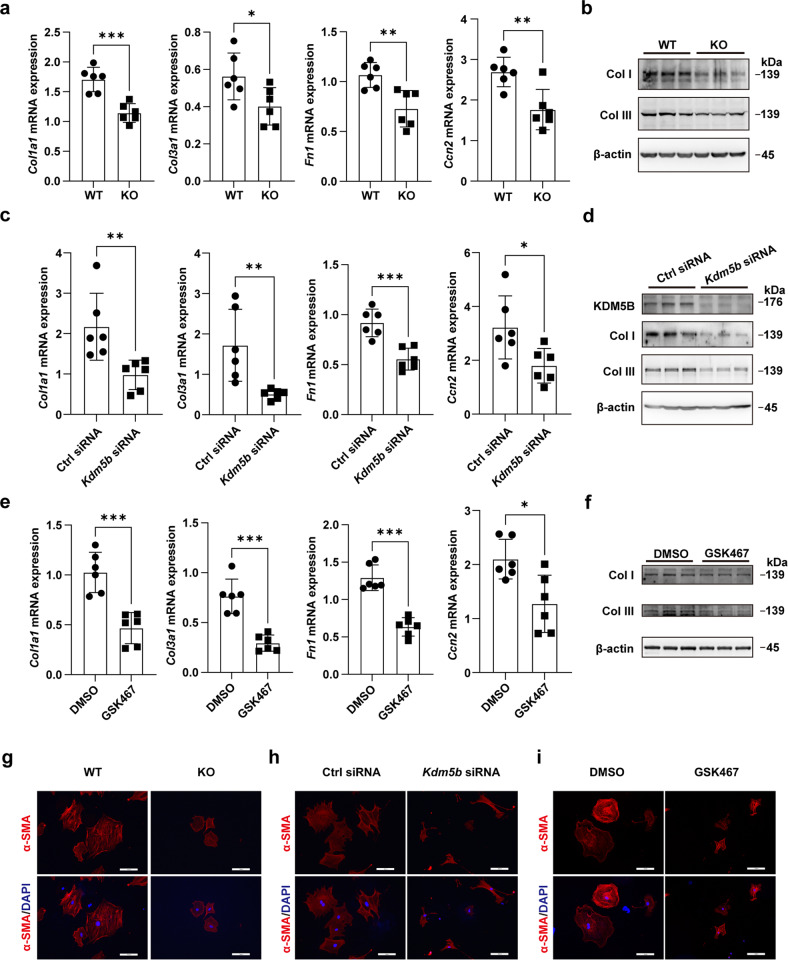
KDM5B promotes fibrotic responses and the transition of cardiac fibroblasts. **a**, **b** Q-PCR analysis of *Col1a1*, *Col3a1*, *Fn1* and *Ccn2* mRNA expression (**a**) (*n* = 6 per group) or immunoblot analysis of Col I and Col III protein expression (**b**) in KDM5B-deficient (KO) or littermate control WT cardiac fibroblasts stimulated with TGF-β (10 ng/ml) for 24 h. **c**, **d** Q-PCR analysis of *Col1a1*, *Col3a1*, *Fn1* and *Ccn2* mRNA expression (**c**) (*n* = 6 per group) or immunoblot analysis of Col I and Col III protein expression (**d**) in *Kdm5b*-silenced or control siRNA-transfected cardiac fibroblasts stimulated with TGF-β (10 ng/ml) for 24 h. **e**, **f** Q-PCR analysis of *Col1a1*, *Col3a1*, *Fn1* and *Ccn2* mRNA expression (**e**) (*n* = 6 per group) or immunoblot analysis of Col I and Col III protein expression (**f**) in cardiac fibroblasts treated with the KDM5B inhibitor GSK467 or DMSO followed by stimulation with TGF-β (10 ng/ml) for 24 h. **g**–**i** Representative immunofluorescence staining of α-SMA (red) in cardiac fibroblasts with KDM5B-deficiency (**g**), KDM5B knockdown (**h**) or GSK467 treatment (**i**) and the corresponding control cardiac fibroblasts (**g**–**i**) stimulated with TGF-β (10 ng/ml) for 24 h. Similar results were obtained from three independent experiments (**g**–**i**). Scale bar, 50 μm. *p < 0.05, **p < 0.01, ***p < 0.001. Unpaired Student’s t test (**a**, **c**, **e**) was performed.

We then investigated the effect of KDM5B on fibrotic responses in fibroblasts. RNA-seq analysis was performed to identify the transcriptome profiling features of primary cardiac fibroblasts isolated from the myocardial tissues of KDM5B-KO and WT mice after MI. Gene set enrichment analysis (GSEA) suggested that KDM5B-KO mice showed decreased expression of genes related to collagen fibril organization and extracellular matrix organization, which are two predominant hallmark features of adverse cardiac remodeling and cardiac fibrosis (Supplementary Fig. 4a–d). Primary cardiac fibroblasts isolated from the myocardial tissues of KDM5B-KO and WT mice were treated with the profibrotic factor TGF-β in vitro. The mRNA expression levels of fibrosis-related genes, including *Col1a1*, *Col3a1*, *Fn1*, and *Ccn2*, were markedly decreased in KDM5B-deficient cardiac fibroblasts in response to TGF-β (Fig. 4a). Consistently, suppressed protein expression levels of collagen I and collagen III were observed in KDM5B-deficient cardiac fibroblasts after TGF-β stimulation (Fig. 4b). Similar decreases in the expression levels of these fibrosis-related molecules were observed in cardiac fibroblasts with *Kdm5b* knockdown mediated by two types of specific siRNAs in response to TGF-β (Fig. 4c, d and Supplementary Fig. 5a–c). GSK467 was identified as a potent and selective inhibitor of KDM5B^20^. After treatment with GSK467, the mRNA levels of *Col1a1*, *Col3a1*, *Fn1*, and *Ccn2* and the protein levels of collagen I and collagen III induced by TGF-β in cardiac fibroblasts were significantly inhibited (Fig. 4e, f). The transformation of fibroblasts to myofibroblasts with a profibrotic phenotype is characterized by increased expression of α-SMA and morphological changes in the formation of numerous actin microfilament bundles^21^. Immunofluorescent staining showed markedly restrained production of α-SMA and preserved fibroblast morphology in KDM5B-deficient cardiac fibroblasts and wild-type fibroblasts with *Kdm5b* knockdown or GSK467 treatment following TGF-β stimulation (Fig. 4g–i and Supplementary Fig. 5d). These results indicate that KDM5B facilitates the formation of myofibroblast phenotypes and fibrosis progression mediated by cardiac fibroblasts.

### KDM5B enhances SMAD-dependent or SMAD-independent TGF-β signaling activation

**Fig. 5:**
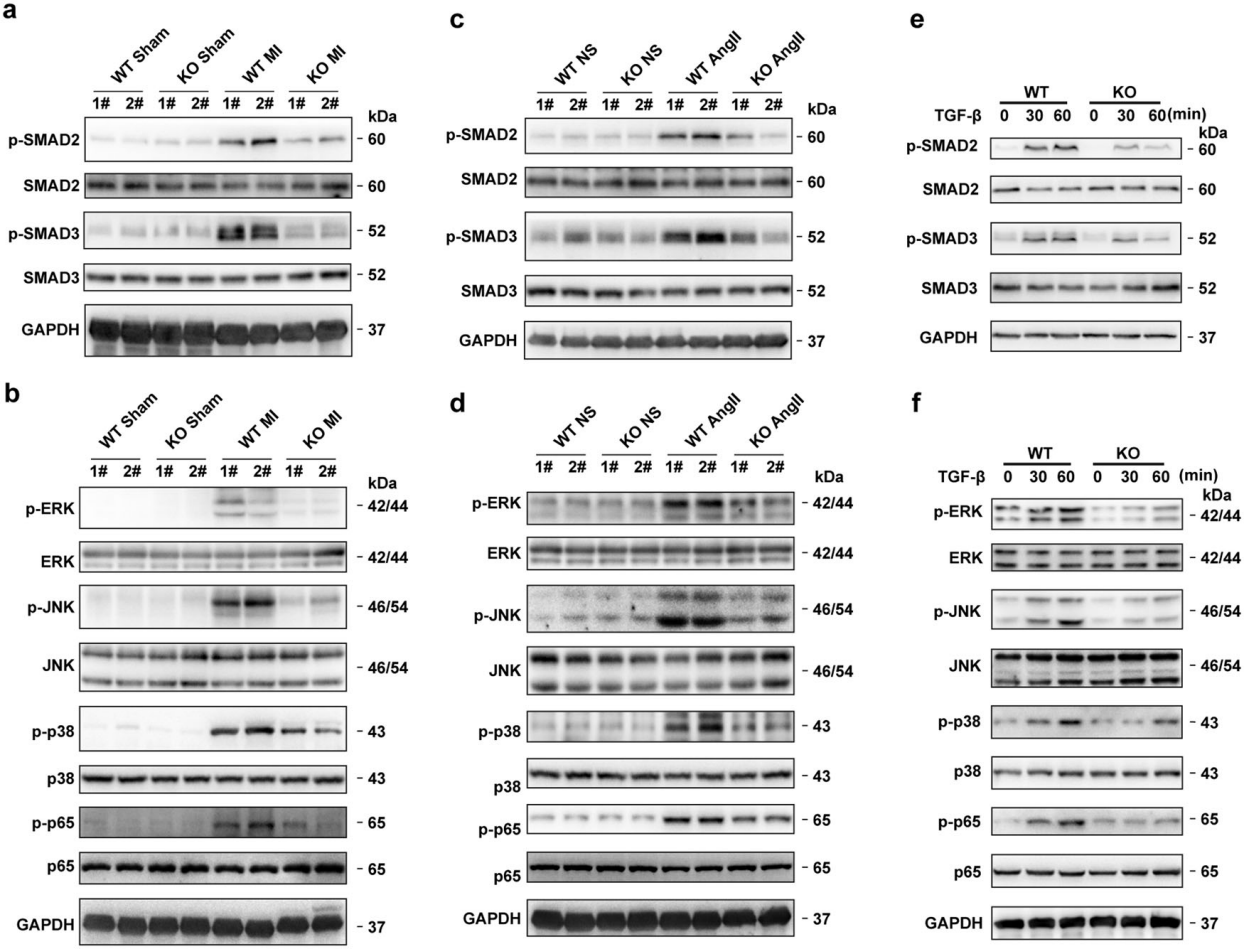
KDM5B facilitates SMAD-dependent or SMAD-independent TGF-β signaling activation. **a** Immunoblot analysis of phosphorylated (p-) or total levels of SMAD2 and SMAD3 proteins in the lysates of myocardial tissues from KDM5B-KO or littermate control WT mice after MI or sham operation. **b** Immunoblot analysis of phosphorylated or total levels of ERK, JNK, p38 and p65 proteins in the lysates of myocardial tissues from KDM5B-KO or WT mice after MI or sham operation. **c** Immunoblot analysis of phosphorylated or total levels of SMAD2 and SMAD3 proteins in the lysates of myocardial tissues from KDM5B-KO or WT mice after AngII or normal saline (NS) infusion. **d** Immunoblot analysis of phosphorylated or total levels of ERK, JNK, p38 and p65 proteins in the lysates of myocardial tissues from KDM5B-KO or WT mice after AngII or normal saline (NS) infusion. **e** Immunoblot analysis of phosphorylated or total levels of SMAD2 and SMAD3 proteins in the lysates of cardiac fibroblasts from KDM5B-KO or WT mice treated with TGF-β (10 ng/ml) for the indicated times. **f** Immunoblot analysis of phosphorylated or total levels of ERK, JNK, p38 and p65 proteins in the lysates of cardiac fibroblasts from KDM5B-KO or WT mice treated with TGF-β (10 ng/ml) for the indicated times. Similar results were obtained from three independent experiments.

Activation of the SMAD-dependent or SMAD-independent TGF-β signaling pathway is an essential determinant of fibrosis. Suppression of SMAD2 and SMAD3 phosphorylation indicated a decrease in the activation of SMAD-dependent TGF-β signaling in the myocardial tissues of KDM5B-KO mice after MI (Fig. 5a). The activation of SMAD-independent TGF-β signaling, including the phosphorylation of ERK, JNK, p38 and p65, was impaired in the myocardial tissues of KDM5B-KO mice after MI (Fig. 5b). Consistently, suppressed activation of SMAD-dependent or SMAD-independent TGF-β signaling, as indicated by decreased phosphorylation of SMAD2, SMAD3, ERK, JNK, p38 and p65, was observed in the myocardial tissues of KDM5B-KO mice following AngII infusion (Fig. 5c, d). Furthermore, KDM5B deficiency or knockdown markedly suppressed the activation of SMAD2, SMAD3, ERK, JNK, p38 and p65 induced by TGF-β in primary cardiac fibroblasts (Fig. 5e–f and Supplementary Fig. 6a, b). These data indicate that KDM5B facilitates fibrotic responses and the transition of cardiac fibroblasts by enhancing SMAD-dependent or SMAD-independent TGF-β signaling activation under pathological conditions.

### The loss of KDM5B promotes angiogenesis through the transition of fibroblasts to endothelial-like cells

**Fig. 6:**
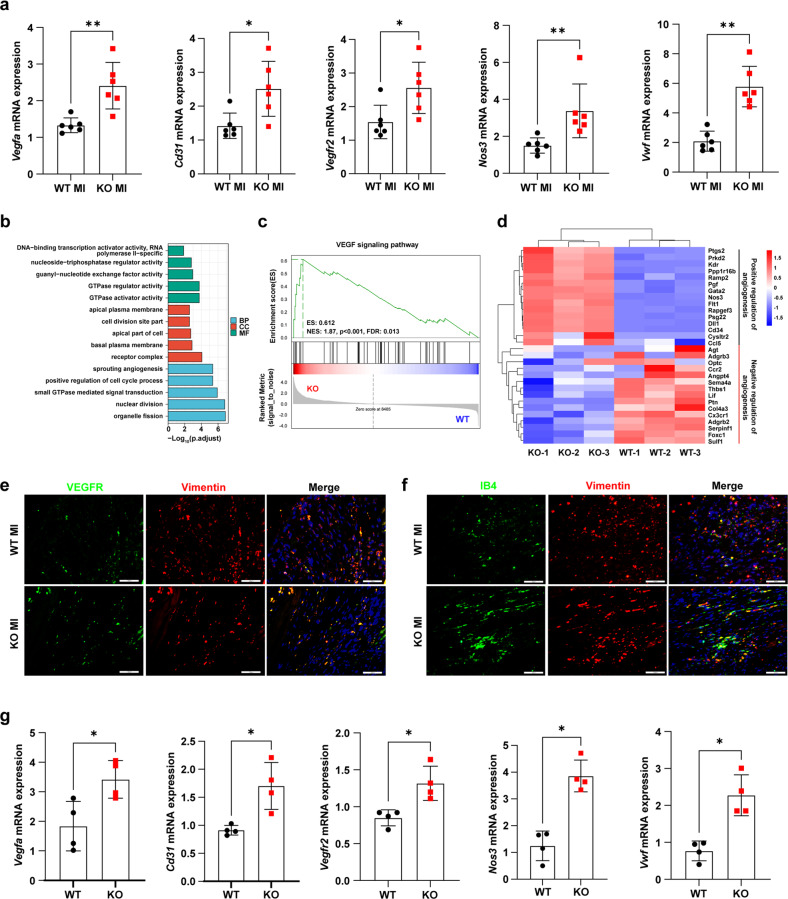
KDM5B deficiency facilitates angiogenesis via the transition of fibroblasts to endothelial-like cells. **a** Q-PCR analysis of *Vegfa*, *Cd31*, *Vegfr2*, *Nos3* and *Vwf* mRNA levels in myocardial tissues from KDM5B-KO or WT mice on Day 7 after MI surgery (*n* = 6 mice per group). **b** Gene Ontology enrichment analysis showing the top 15 differentially expressed genes in primary cardiac fibroblasts isolated from the heart tissues of KDM5B-KO and littermate control WT mice on Day 7 after MI surgery (BP biological process, CC cellular component, MF molecular function). **c** GSEA enrichment plot showing gene sets associated with the VEGF signaling pathway in primary cardiac fibroblasts as in **b**. **d** Heatmap showing differentially expressed genes relative to the positive and negative regulation of angiogenesis in primary cardiac fibroblasts as in **b**. **e**, **f** Representative immunofluorescence staining of VEGFR (green) (**e**), IB4 (green) (**f**) and Vimentin (red) (**e**, **f**) in myocardial tissues from KDM5B-KO or WT mice on Day 7 after MI surgery. Scale bar, 50 μm. Similar results were obtained from three independent experiments. **g** Q-PCR analysis of *Vegfa*, *Cd31*, *Vegfr2*, *Nos3* and *Vwf* mRNA expression in KDM5B-deficient (KO) or WT cardiac fibroblasts stimulated with TGF-β (10 ng/ml) for 24 h (*n* = 4 per group). *p < 0.05, **p < 0.01. Unpaired Student’s *t* test (**a**, **g**) was performed.

Previous studies have demonstrated that angiogenesis ameliorates cardiac dysfunction and adverse cardiac remodeling after MI and AngII-induced pressure overload^22,23^. Microarray analysis of RNA from the myocardial tissues of KDM5B-KO mice and control littermate WT mice after MI indicated the enriched differentially expressed genes linked to angiogenesis (Supplementary Fig. 7a). Furthermore, the increased expression of positively regulated angiogenesis-associated genes and decreased expression of negatively regulated angiogenesis-associated genes were observed in the myocardial tissues of KDM5B-KO mice after MI (Supplementary Fig. 7b). KDM5B deficiency enhanced the expression levels of endothelial marker genes, including vascular endothelial growth factor A (*Vegfa*), *Cd31*, vascular endothelial growth factor receptor 2 (*Vegfr2*), nitric oxide synthase 3 (*Nos3*) and von Willebrand factor (*Vwf*), in myocardial tissues after MI (Fig. 6a). Angiogenesis mediated by the fibroblast-to-endothelial cell transition has been reported to facilitate cardiac repair after myocardial injury^9^. Consistent with the microarray analysis, further RNA-seq analysis of fibroblasts isolated from the myocardial tissues of KDM5B-KO and WT mice showed the enrichment of differentially expressed genes related to angiogenesis after MI (Fig. 6b). GSEA showed a significantly upregulated gene set enriched in the widely recognized proangiogenic VEGF signaling pathway in cardiac fibroblasts from KDM5B-KO mice compared with WT mice after MI (Fig. 6c). Furthermore, the top differentially expressed genes revealed were the increased expression of positively regulated angiogenesis-related genes and decreased expression of negatively regulated angiogenesis-related genes in cardiac fibroblasts from KDM5B-KO mice after MI (Fig. 6d). In addition, cardiomyocytes and cardiac fibroblasts were isolated from the myocardial tissues of KDM5B-KO and WT mice on Day 7 after MI for gene alteration detection. As shown in Supplementary Fig. 7c, KDM5B deficiency did not affect angiogenesis-related gene expression in cardiomyocytes but increased their expression levels in cardiac fibroblasts. Immunofluorescence staining showed increased expression of VEGFR or IB4 colocalized with Vimentin-positive cardiac fibroblasts in the myocardial tissues of KDM5B-KO mice after MI (Fig. 6e, f and Supplementary Fig. 7d, e). KDM5B deficiency increased the expression levels of endothelial marker genes, including *Vegfa*, *Cd31*, *Vegfr2*, *Nos3* and *Vwf*, in primary cardiac fibroblasts isolated from the myocardial tissues of KDM5B-KO mice (Fig. 6g). KDM5B deficiency enhanced tube formation mediated by primary cardiac fibroblasts (Supplementary Fig. 7f). These data indicate that the loss of KDM5B facilitates the transition of fibroblasts to endothelial-like cells and reinforces angiogenesis, attenuating cardiac fibrosis and dysfunction in response to pathological injuries.

### KDM5B exacerbates cardiac fibrosis by suppressing ATF3 expression

**Fig. 7:**
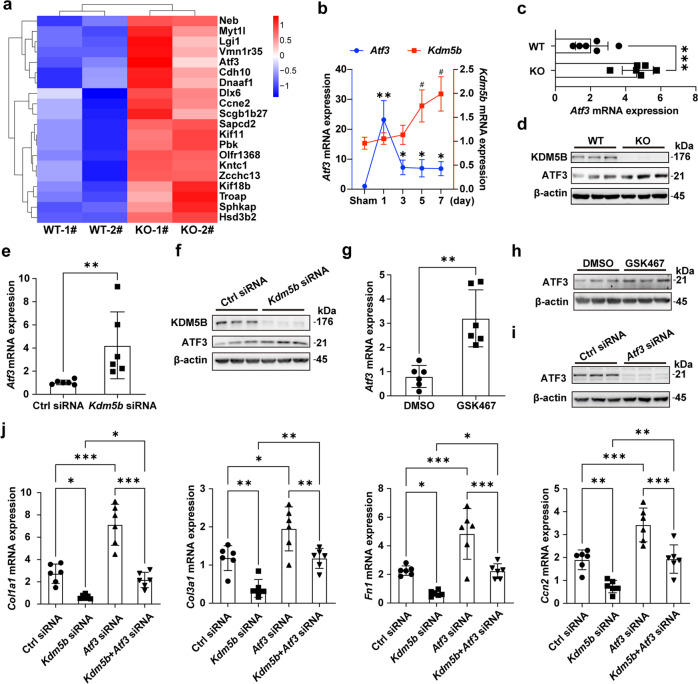
KDM5B promotes cardiac fibrosis by inhibiting ATF3 expression. **a** Heatmap showing the top 20 upregulated differentially expressed genes in myocardial tissues from KDM5B-KO and littermate control WT mice on Day 7 after MI surgery. **b** Q-PCR analysis of *Atf3* and *Kdm5b* mRNA expression in the myocardial tissues of WT mice on the indicated day after MI or sham operation (*n* = 6 per group). ^#^*p* < 0.05 vs. *Kdm5b* mRNA levels in the sham operation group; *p < 0.05, **p < 0.01 vs. *Atf3* mRNA levels in the sham operation group. **c**, **d** Q-PCR analysis of *Atf3* mRNA (**c**) (*n* = 6 mice per group) or immunoblot analysis of ATF3 protein levels (**d**) in myocardial tissues from KDM5B-KO or WT mice on Day 7 after MI surgery. **e**, **f** Q-PCR analysis of *Atf3* mRNA expression (**e**) (*n* = 6 per group) or immunoblot analysis of ATF3 protein expression (**f**) in *Kdm5b*-silenced or control siRNA-transfected cardiac fibroblasts stimulated with TGF-β (10 ng/ml) for 24 h. **g**, **h** Q-PCR analysis of *Atf3* mRNA expression (**g**) (*n* = 6 per group) or immunoblot analysis of ATF3 protein expression (**h**) in cardiac fibroblasts treated with the KDM5B inhibitor GSK467 or DMSO followed by stimulation with TGF-β (10 ng/ml) for 24 h. **i** Immunoblot analysis of ATF3 protein expression in cardiac fibroblasts transfected with *Atf3* siRNA or control siRNA. **j** Q-PCR analysis of *Col1a1*, *Col3a1*, *Fn1* and *Ccn2* mRNA expression levels in cardiac fibroblasts transfected with *Kdm5b* siRNA, *Atf3* siRNA, control siRNA or cotransfected with *Kdm5b* and *Atf3* siRNA followed by stimulation with TGF-β (10 ng/ml) for 24 h (*n* = 6 per group). *p < 0.05, **p < 0.01, ***p < 0.001. Unpaired Student’s *t* test (**c**, **e**, **g**) and ANOVA (**b**, **j**) were performed.

We further investigated the mechanism underlying the attenuated cardiac fibrosis and dysfunction mediated by KDM5B deficiency. Among the top upregulated expressed genes screened from RNAs of myocardial tissues in KDM5B-KO mice and control littermate WT mice after MI, the marked increased expression of *Atf3* in myocardial tissues of KDM5B-KO mice raised our attention (Fig. 7a). Consistently, RNA-seq analysis showed increased expression of *Atf3* in KDM5B-deficient cardiac fibroblasts (Supplementary Fig. 8a). Previous studies have shown that elevated ATF3 expression in cardiac fibroblasts can prevent adverse cardiac remodeling and suppress myocardial fibrosis induced by ischemic or hypertrophic injury^24,25^. *Atf3* expression in the myocardial tissues of wild-type mice peaked on Day 1 after MI and then decreased to a relatively higher level on Days 3-7 (Fig. 7b). KDM5B deficiency increased the mRNA and protein expression of ATF3 in myocardial tissues after MI (Fig. 7c, d). The mRNA and protein levels of ATF3 in primary cardiac fibroblasts stimulated with TGF-β were upregulated by *Kdm5b* knockdown or GSK467 inhibitor treatment (Fig. 7e–h and Supplementary Fig. 8b, c). The expression levels of fibrosis-related genes such as *Col1a1*, *Col3a1*, *Fn1* and *Ccn2* in primary cardiac fibroblasts induced by TGF-β were significantly decreased after *Kdm5b* knockdown or were increased after *Atf3* knockdown alone, while the reduced expression of these fibrotic genes mediated by *Kdm5b* knockdown could be reversed by knockdown of *Atf3* (Fig. 7i, j). These data indicate that ATF3 is a downstream target of KDM5B and that KDM5B deficiency attenuates cardiac fibrosis by increasing ATF3 expression.

### KDM5B represses ATF3 expression through histone demethylation of the *Atf3* promoter

**Fig. 8:**
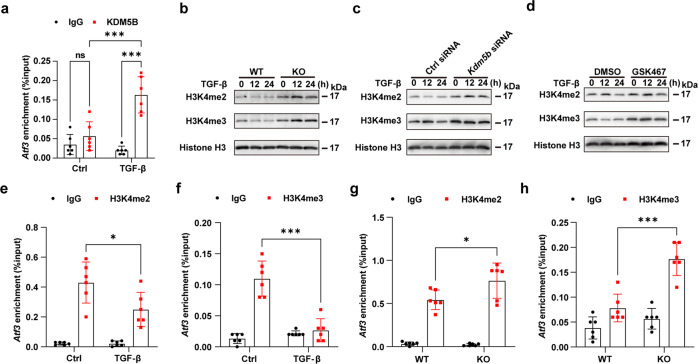
KDM5B suppresses ATF3 expression through histone demethylation of the *Atf3* promoter. **a** ChIP analysis of KDM5B enrichment levels at the *Atf3* promoter in WT cardiac fibroblasts treated with TGF-β (10 ng/ml) or the control PBS (Ctrl) for 24 h. *n* = 6 per group. **b**–**d** Immunoblot analysis of H3K4me2 and H3K4me3 levels in cardiac fibroblasts with KDM5B deficiency (**b**), KDM5B knockdown (**c**) or GSK467 treatment (**d**) and the corresponding control cardiac fibroblasts (**b**–**d**) stimulated with TGF-β (10 ng/ml) for the indicated times. Similar results were obtained from three independent experiments. **e**, **f** ChIP analysis of H3K4me2 (**e**) or H3K4me3 (**f**) levels at the *Atf3* promoter of WT cardiac fibroblasts treated with TGF-β (10 ng/ml) or control PBS (Ctrl) for 24 h. *n* = 6 per group. **g**, **h** ChIP analysis of H3K4me2 (**g**) or H3K4me3 (**h**) levels at the *Atf3* promoter of KDM5B-deficient (KO) or WT cardiac fibroblasts stimulated with TGF-β (10 ng/ml) for 24 h. *n* = 6 per group. *p < 0.05, ***p < 0.001. Two-way ANOVA (**a**, **e**–**h**) was performed.

Since KDM5B is a histone H3K4me3 and H3K4me2 demethylase^15^, we next explored whether KDM5B regulated ATF3 expression by affecting the methylation level of histone H3K4. The recruitment of KDM5B to the *Atf3* promoter was markedly enhanced in cardiac fibroblasts after TGF-β stimulation (Fig. 8a). Markedly increased H3K4me2 and H3K4me3 levels were observed in cardiac fibroblasts isolated from the myocardial tissues of KDM5B-KO mice (Fig. 8b). Consistently, *Kdm5b* knockdown or GSK467 treatment elevated the levels of H3K4me2 and H3K4me3 in wild-type cardiac fibroblasts stimulated with TGF-β (Fig. 8c, d). Decreased levels of H3K4me2 and H3K4me3 at the promoter of the *Atf3* gene were found in wild-type cardiac fibroblasts after stimulation with TGF-β for 24 h (Fig. 8e, f). In contrast, KDM5B deficiency notably increased the levels of H3K4me2 and H3K4me3 at the promoter of the *Atf3* gene in cardiac fibroblasts in response to TGF-β treatment (Fig. 8g, h). These results demonstrate that KDM5B directly binds to the *Atf3* promoter and inhibits ATF3 transcription via its histone demethylase activity, which in turn exacerbates cardiac fibrosis and adverse cardiac remodeling following MI and pressure overload (Supplementary Fig. 9).

## Discussion

Cardiac fibrosis, which is the most common pathological feature of many cardiac disorders, is associated with poor prognosis, such as heart failure. In this study, we revealed the novel role of the histone demethylase KDM5B in facilitating the pathogenesis of cardiac fibrosis. KDM5B mediated the demethylation of activated H3K4me2/3 on the *Atf3* promoter and repressed *Atf3* expression, resulting in excessive cardiac fibrosis and the deterioration of cardiac function following ischemic and hypertrophic attack.

Aberrant expression of multiple pathogenic genes is closely associated with the development and progression of cardiac diseases. The distinct epigenetic modifications and sites of histones play critical and precise roles in regulating the transcription of these pathogenic genes^26^. Among the different types of histone modifications, the reversible methylation modification profile mediated by histone methyltransferases and demethylases has important functions in multiple physiological and pathological processes^27^. A previous study showed that G9a, a methyltransferase that mediates mono-/di-methylation of H3K9, formed a complex with myocyte enhancer factor 2C (MEF2C) and bound heterochromatin to suppress cardiac fibrosis^28^. Silencing of the endothelial-specific histone methyltransferase SET domain containing 1 (SET1) could inhibit cardiac fibrosis and hypertrophy by repressing endothelin-1 transcription in response to chronic AngII treatment^29^. However, studies on the roles of histone demethylases in pathological cardiac fibrosis are limited. Myocardin-related transcription factor A (MRTF-A) promotes macrophage trafficking by interacting with lysine demethylase 3 A (KDM3A) to worsen cardiac fibrosis and hypertrophy, but whether KDM3A functions in a demethylase activity-dependent manner remains unclear^30^. For the important histone H3K4me2/3 demethylase KDM5B, previous studies on its function have focused on oncological and infectious diseases. For example, KDM5B suppressed antitumor immunity and mediated immune evasion in melanoma by recruiting SETDB1 (SET domain bifurcated histone lysine methyltransferase 1) to silence retroelements^31^. KDM5B deficiency significantly promoted the production of inflammatory cytokines and facilitated innate inflammatory responses triggered by diverse pathogens^17,32^. Our study provides evidence that KDM5B drives the progression of pathological cardiac fibrosis by mediating the demethylation of activated H3K4me2/3 on the *Atf3* promoter and inhibiting its expression. Thus, our findings identify KDM5B as a novel profibrotic histone demethylase and reveal a new epigenetic regulatory approach for cardiac fibrosis under pathological stress conditions.

The main manifestations of cardiac fibrosis are the excessive conversion of fibroblasts into myofibroblasts accompanied by excessive synthesis and insufficient degradation of the extracellular matrix, which leads to decreased myocardial compliance, diastolic-systolic dysfunction and even the onset of heart failure^2^. Pathological cardiac fibrosis is dependent on the transcriptional regulation of multiple fibrotic signaling pathways, of which transcription factors have important regulatory roles in the activation of signal components^33^. In this study, we found that ATF3 expression was markedly increased in the myocardium of KDM5B-deficient mice under pathological conditions. We further demonstrated that KDM5B deficiency promoted ATF3 expression by elevating the levels of activated H3K4me2/3 methylation at its promoter region. These results confirm that the transcription factor ATF3 is an essential downstream target of KDM5B during cardiac fibrosis. Previous studies have shown that ATF3 plays dual roles in fibrosis in different organs. Accumulating evidence has demonstrated that ATF3 limits cardiac fibrosis progression and prevents adverse cardiac remodeling triggered by ischemic or hypertrophic injury^24,25,34^. However, ATF3 overexpression was found in fibrotic livers and contributed to liver fibrosis by activating hepatic stellate cells^35^. Furthermore, ATF3 plays a profibrotic role in systemic sclerosis and bleomycin-induced lung injury^36,37^. The functional differences in ATF3 in organ fibrosis may be associated with different cell types and profibrotic mechanisms. Consistent with previous reports on the heart, our data confirm that ATF3, which is a key regulator that is epigenetically suppressed by KDM5B, can inhibit myocardial fibrosis mediated by cardiac fibroblasts in response to ischemic or hypertrophic stress.

Due to the extremely low regeneration capacity of cardiomyocytes, the reconstruction and restoration of the blood flow network in the infarcted area are critical for repair after myocardial injury in ischemic diseases. Cardiac angiogenesis is crucial for maintaining cardiac function and myocardial adaptation to pressure overload^23,38^. Endothelial cells are considered to be the most important cells that mediate angiogenesis, but the contribution of fibroblasts should not be neglected. Fibroblasts can promote endothelial cell-mediated angiogenesis through paracrine secretion and can transdifferentiate into endothelial cells, mediating angiogenesis after cardiac injury^9,39–41^. Our study provides evidence that KDM5B deficiency increases the expression of angiogenesis-associated genes and promotes the transdifferentiation of fibroblasts to endothelial-like cells, which enhances angiogenesis and contributes to the alleviation of cardiac dysfunction under pathological conditions. The underlying mechanism of enhanced angiogenesis mediated by KDM5B deficiency may involve regulating the expression of several transcription factors, such as Nodal, which needs to be further investigated.

Epigenetic therapies have emerged as novel and valuable areas of research in drug discovery. The availability of various regulators of epigenetic modification enzymes in clinical trials has mostly focused on oncological diseases. For example, the selective inhibitor of the histone methyltransferase EZH2 (enhancer of zeste 2 polycomb repressive complex 2 subunit) or histone demethylase LSD1 (lysine-specific demethylase 1) showed promising antitumor activity in solid tumors or leukemia^42,43^. However, fewer epigenetic inhibitors have shown effective responses in clinical trials for diseases other than cancers, especially cardiovascular diseases. Our in vitro results confirmed the effectiveness of the KDM5B inhibitor in suppressing fibroblast-mediated profibrotic responses. Together with the in vivo data showing the attenuation of cardiac fibrosis mediated by KDM5B deficiency, our study opens new paths for the development of KDM5B-targeting epigenetic therapy to overcome pathological cardiac fibrosis.

In summary, our results demonstrate that the histone demethylase KDM5B suppresses ATF3 expression by demethylating H3K4me2/3 on its promoter, subsequently promoting excessive expression of profibrotic genes and inhibiting angiogenesis. KDM5B drives the progression of cardiac fibrosis and facilitates adverse cardiac remodeling in response to ischemic or hypertrophic stress, which will aid in the development of novel epigenetic therapies for pathological cardiac fibrosis and heart failure.

## Supplementary information

Supplementary information
