# Supplementary material for: Loss of KDM5B ameliorates pathological cardiac fibrosis and dysfunction by epigenetically enhancing ATF3 expression
Source: Exp Mol Med. 2022 Dec 8;54(12):2175–87. doi: 10.1038/s12276-022-00904-y (PMC9794816; doi:10.1038/s12276-022-00904-y)
Supplement: Supplementary file 1 — Supplementary information [file 12276_2022_904_MOESM1_ESM.docx]

**Supplementary information**

**Loss of** **KDM5B ameliorates pathological cardiac fibrosis and dysfunction by epigenetically enhancing ATF3 expression**

Bo Wang, Yong Tan, Yunkai Zhang, Sheng Zhang, Xuewen Duan, Yuyu Jiang, Tong Li, Qingqing Zhou, Xingguang Liu, Zhenzhen Zhan

**
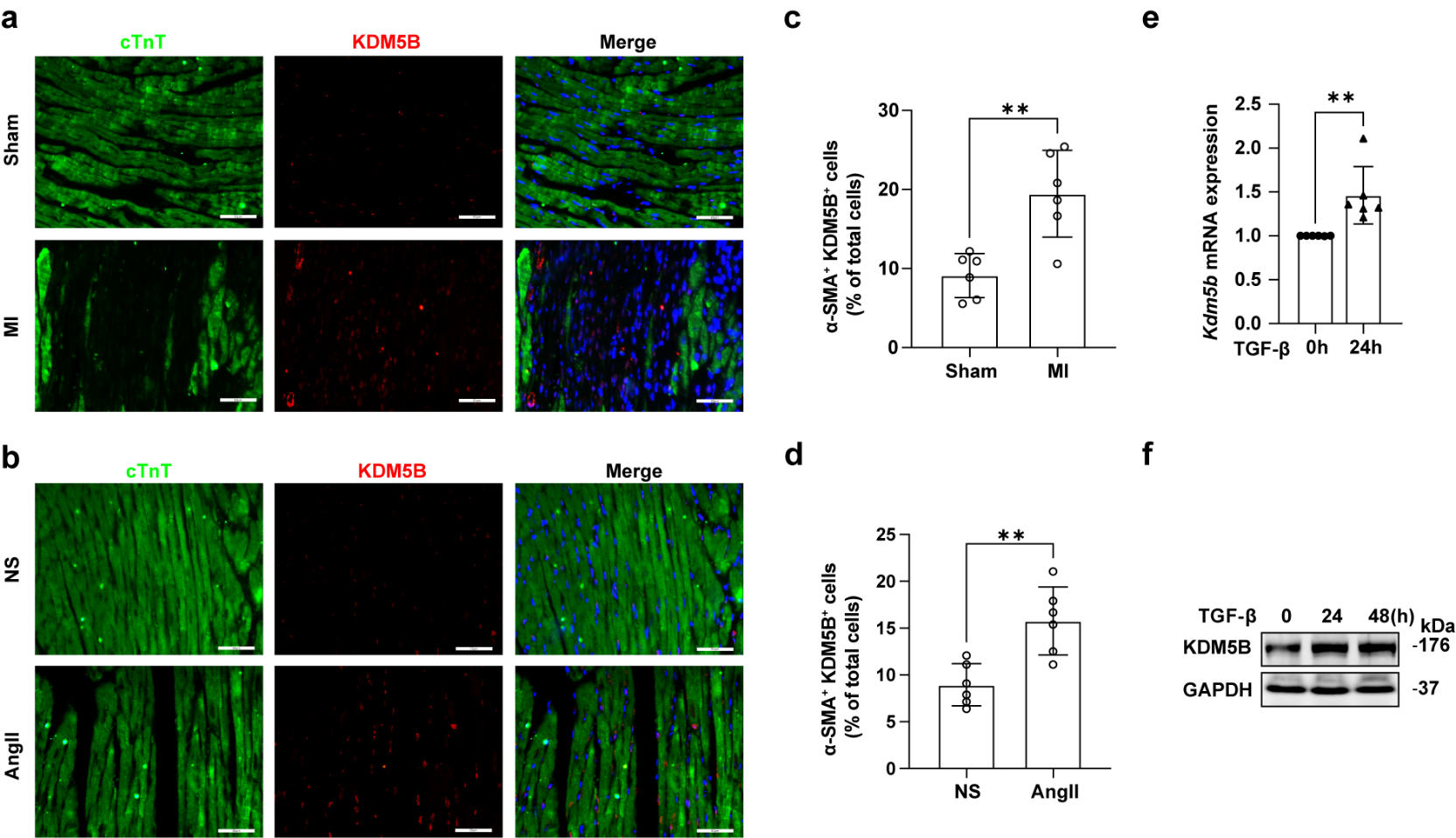
**

**Supplementary Fig. 1 KDM5B is upregulated in cardiac fibroblasts after TGF-β stimulation.**

**a** Representative immunofluorescence staining of KDM5B (red) or cTnT (green) of myocardial tissues from wild type mice at day 7 after MI or sham operation. Scale bar, 50 μm. **b** Representative immunofluorescence staining of KDM5B (red) or cTnT (green) of myocardial tissues from wild type mice at day 28 after AngII or NS infusion. Scale bar, 50 μm. **c** Quantitation of α-SMA and KDM5B positive cells through immunofluorescence staining of myocardial tissues from wild type mice at day 7 after MI or sham operation. (n = 6 mice per group; relative to Fig. 1e). **d** Quantitation of α-SMA and KDM5B positive cells through immunofluorescence staining of myocardial tissues from wild type mice at day 28 after AngII or NS infusion. (n = 6 mice per group; relative to Fig. 1f). **e**-**f** Q-PCR analysis of *Kdm5b* mRNA (e) or immunoblot analysis of KDM5B protein (f) expression in wild type cardiac fibroblasts stimulated with TGF-β (10 ng/ml) for the indicated times (n = 6 per group). Similar data were from three independent experiments. ***p* < 0.01, unpaired Student's t-test (c-e) was performed.


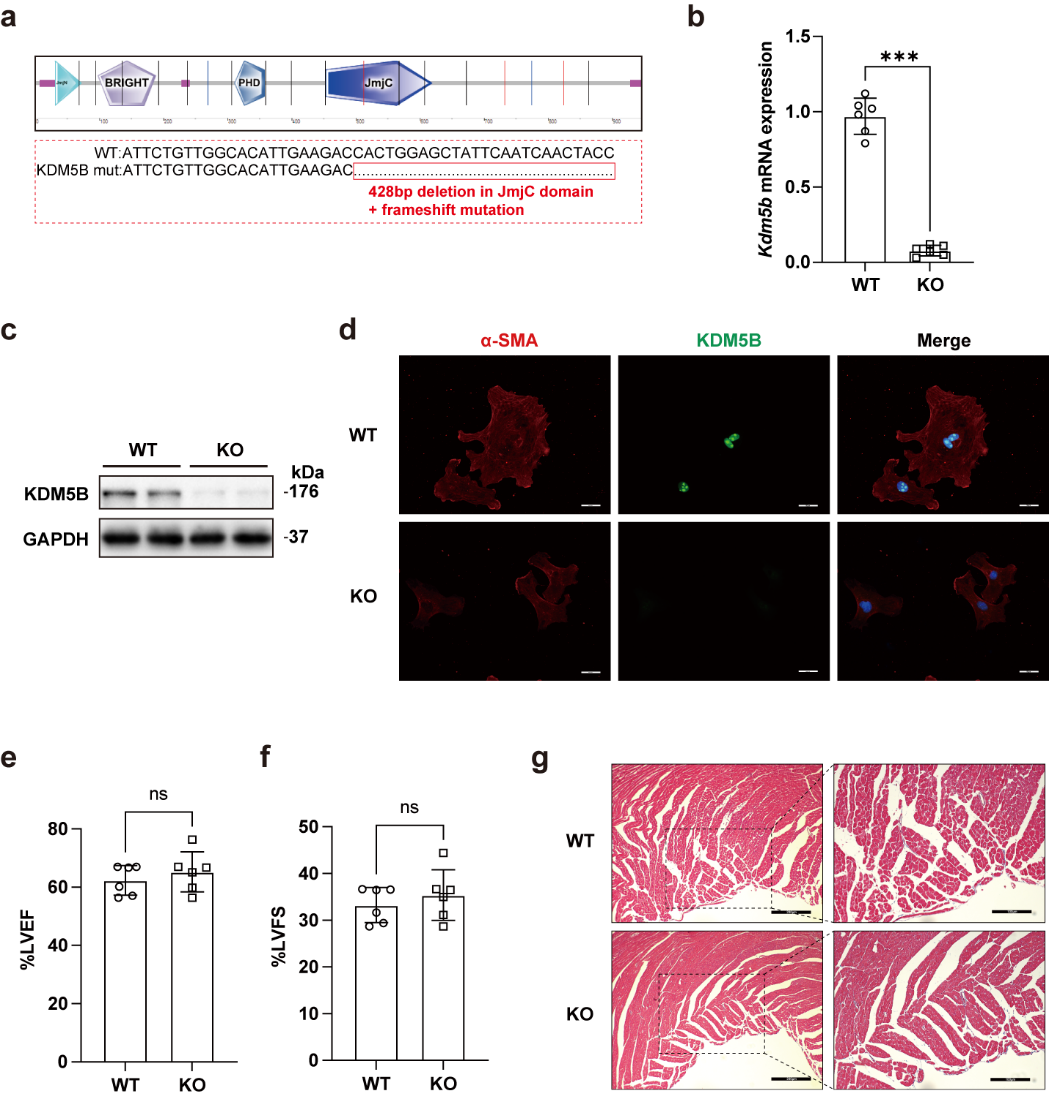


**Supplementary Fig. 2 KDM5B deficiency does not affect cardiac function in physiological conditions.**

**a** Schematic diagram of targeted disruption of *Kdm5b* gene with the CRISPR/Cas9 system. **b**-**c** Q-PCR analysis of *Kdm5b* mRNA (b) (n = 6 mice per group) or immunoblot analysis of KDM5B protein (c) expression in cardiac fibroblasts isolated from KDM5B KO or WT mice. **d** Representative immunofluorescence staining of α-SMA (red) and KDM5B (green) in cardiac fibroblasts isolated from KDM5B KO or WT mice stimulated with TGF-β (10 ng/ml) for 24 h. Scale bar, 50 μm. **e**-**f** Echocardiographic measurement of LVEF and LVFS of KDM5B KO or WT mice at physiological conditions. **g** Representative Masson's Trichrome staining images in myocardial tissues from KDM5B KO or WT mice at physiological conditions. Scale bar, 200 μm (upper), 100 μm (bottom). ****p* < 0.001, unpaired Student's t-test (b, e, f) was performed.


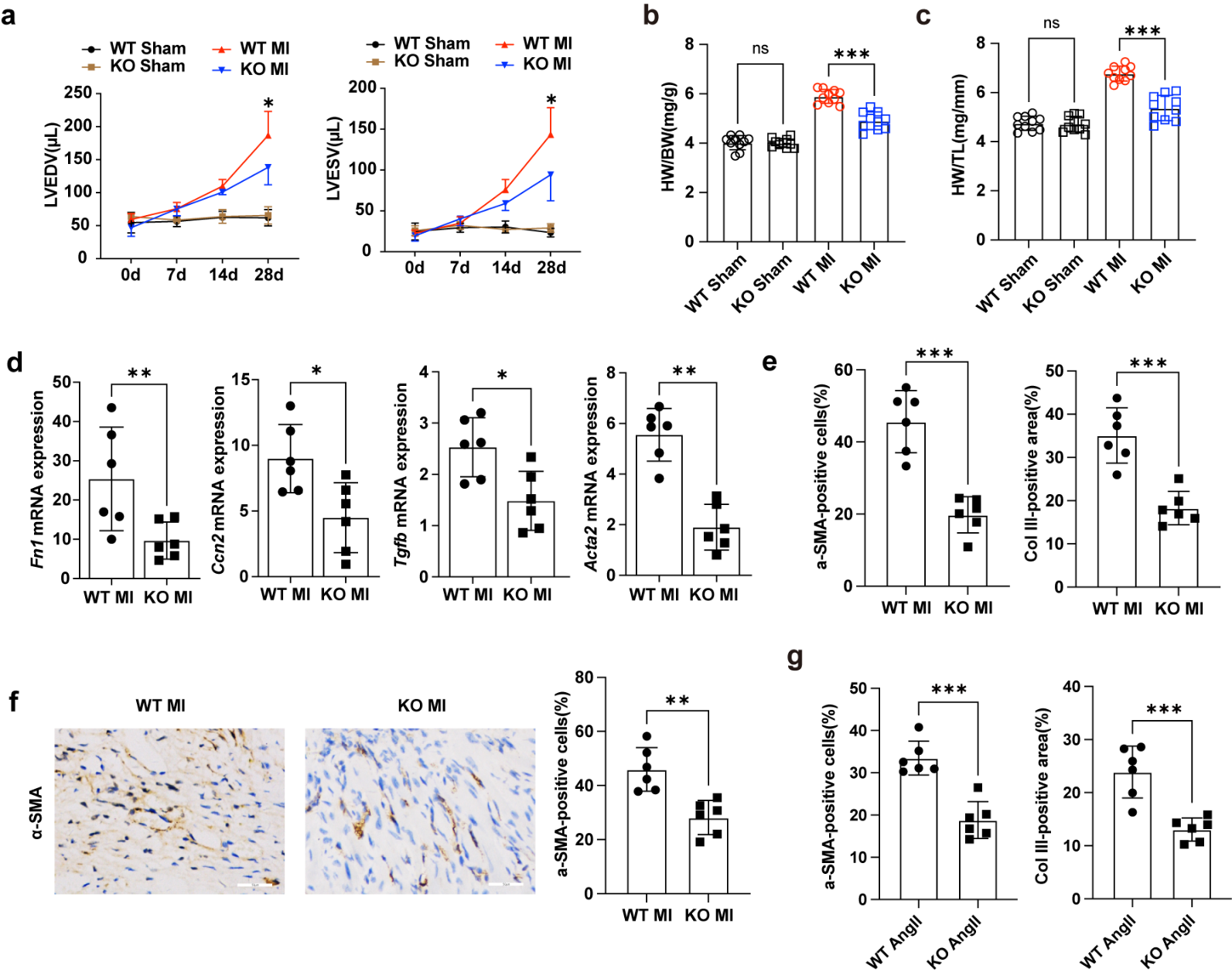


**Supplementary Fig. 3 KDM5B deficiency prevents MI-induced cardiac dysfunction and cardiac fibrosis.**

**a** Echocardiographic measurement of left ventricular end-diastolic volume (LVEDV) and left ventricular end-systolic volume (LVESV) of KDM5B KO or littermate control WT mice at baseline (day 0) and the indicated day after MI or sham operation (n = 6 mice per group). **b-c** Ratios of HW/BW (b) and HW/TL (c) (n = 10 mice per group) from KDM5B KO or WT mice at day 14 after MI or sham operation. **d** Q-PCR analysis of *Fn1*, *Ccn2*, *Tgfb* and *Acta2* mRNA levels in myocardial tissues from KDM5B KO or WT mice at day 14 post-MI (n = 6 mice per group). **e** Quantitation of α-SMA-positive or Col III-positive cells through immunofluorescence staining in myocardial tissues from KDM5B KO or WT mice at day 14 post-MI (n = 6 per group; relative to Fig. 2g). **f** Representative immunohistochemistry staining of α-SMA and quantitation of α-SMA-positive cells in myocardial tissues from KDM5B KO or WT mice at day 14 post-MI. n = 6 per group. Scar bar, 50 μm. **g** Quantitation of α-SMA-positive or Col III-positive cells through immunofluorescence staining in myocardial tissues from KDM5B KO or WT mice at day 28 after AngⅡ infusion. (n = 6 per group; relative to Fig. 3l). **p* < 0.05, ***p* < 0.01, ****p* < 0.001. Two-way ANOVA (a-c) or unpaired Student's t-test (d-g) was performed.


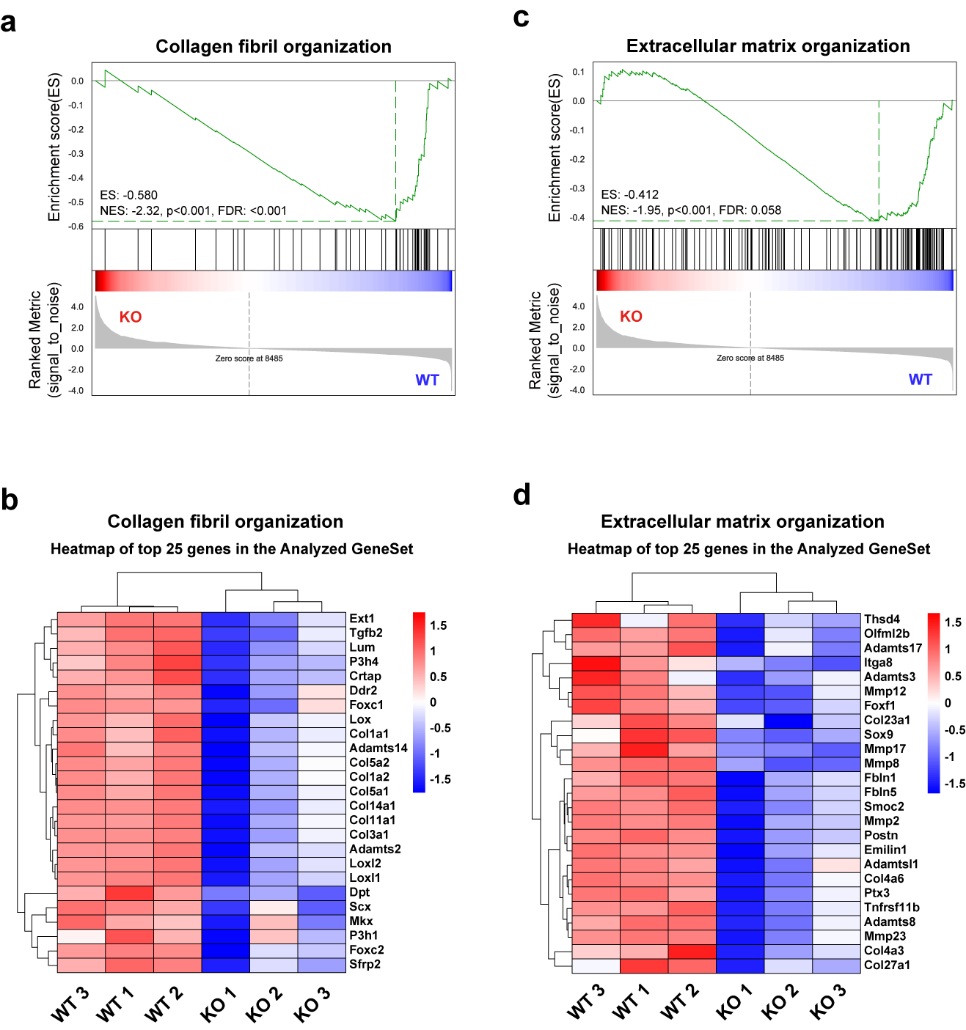


**Supplementary Fig. 4 KDM5B deficiency restrains the expression of adverse cardiac remodeling-related genes after MI.**

**a** and **c** GSEA enrichment plots of gene sets associated with the collagen fibril organization and extracellular matrix organization in cardiac fibroblasts isolated from heart tissues of KDM5B KO or WT mice at day 7 after MI. **b** and **d** Heatmap showing selected top 25 differentially expressed genes associated with the collagen fibril organization and extracellular matrix organization in cardiac fibroblasts isolated from heart tissues of KDM5B KO or WT mice at day 7 after MI.


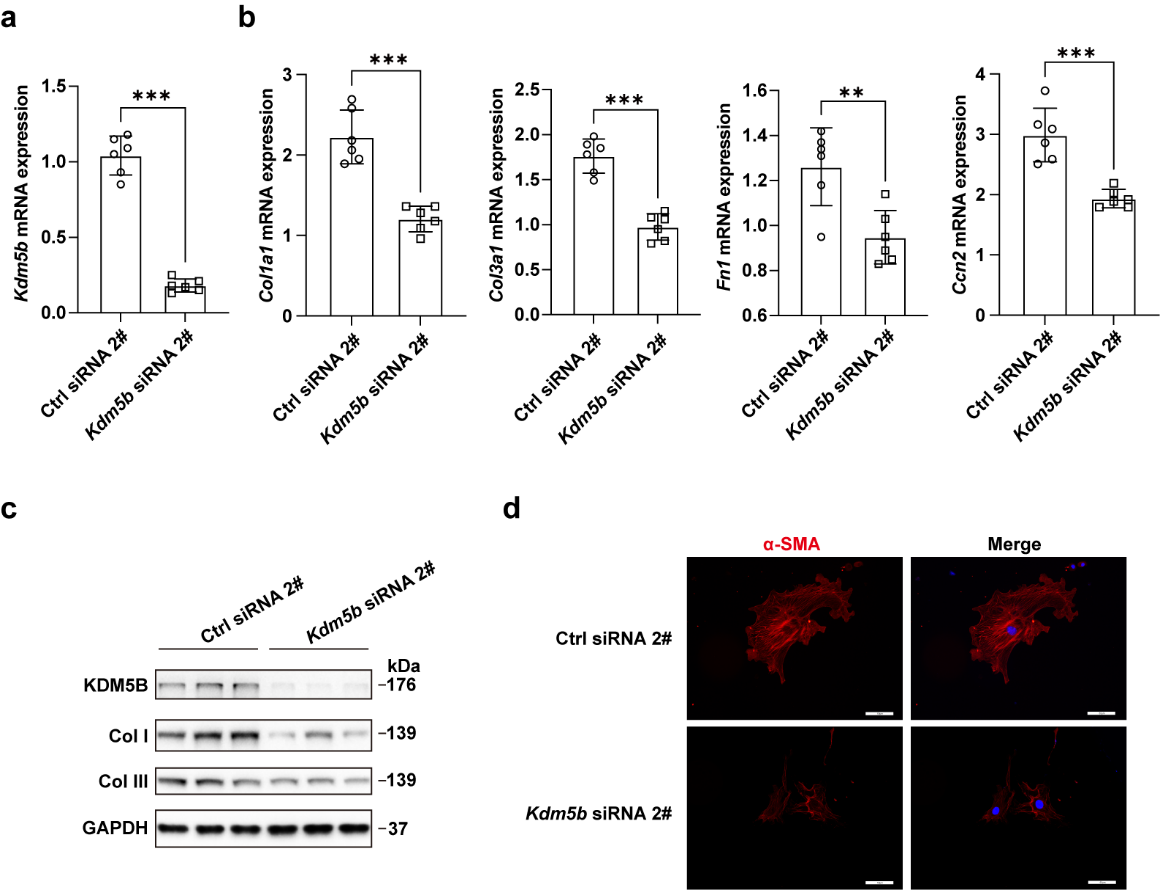


**Supplementary Fig. 5 KDM5B knockdown suppresses fibrotic responses and transition of cardiac fibroblasts.**

**a** Q-PCR analysis of *Kdm5b* mRNA expression in cardiac fibroblasts transfected with *Kdm5b* siRNA or control siRNA (n = 6 per group). **b** Q-PCR analysis of *Col1a1*, *Col3a1*, *Fn1* and *Ccn2* mRNA expression in *Kdm5b*-silenced or control siRNA-transfected cardiac fibroblasts stimulated with TGF-β (10 ng/ml) for 24 h (n = 6 per group). **c** Immunoblot analysis of KDM5B, Col Ⅰ and Col Ⅲ protein expression in *Kdm5b*-silenced or control siRNA-transfected cardiac fibroblasts stimulated with TGF-β (10 ng/ml) for 24 h. **d** Representative immunofluorescence staining of α-SMA (red) in cardiac fibroblasts transfected with *Kdm5b* siRNA or control siRNA stimulated with TGF-β (10 ng/ml) for 24 h. Scale bar, 50 μm. Similar results were obtained from three independent experiments (c, d). ***p* < 0.01, ****p* < 0.001. Unpaired Student's t-test (a, b) was performed.





**Supplementary Fig. 6 KDM5B knockdown restrains SMAD-dependent or -independent TGF-β signaling activation.**

**a** Immunoblot analysis of phosphorylation or total levels of SMAD2 and SMAD3 proteins in lysates of *Kdm5b*-silenced or control siRNA-transfected cardiac fibroblasts treated with TGF-β (10 ng/ml) for the indicated times. **b** Immunoblot analysis of phosphorylation or total levels of ERK, JNK, p38 and p65 proteins in lysates of *Kdm5b*-silenced or control siRNA-transfected cardiac fibroblasts treated with TGF-β (10 ng/ml) for the indicated times. Similar results were obtained from three independent experiments.


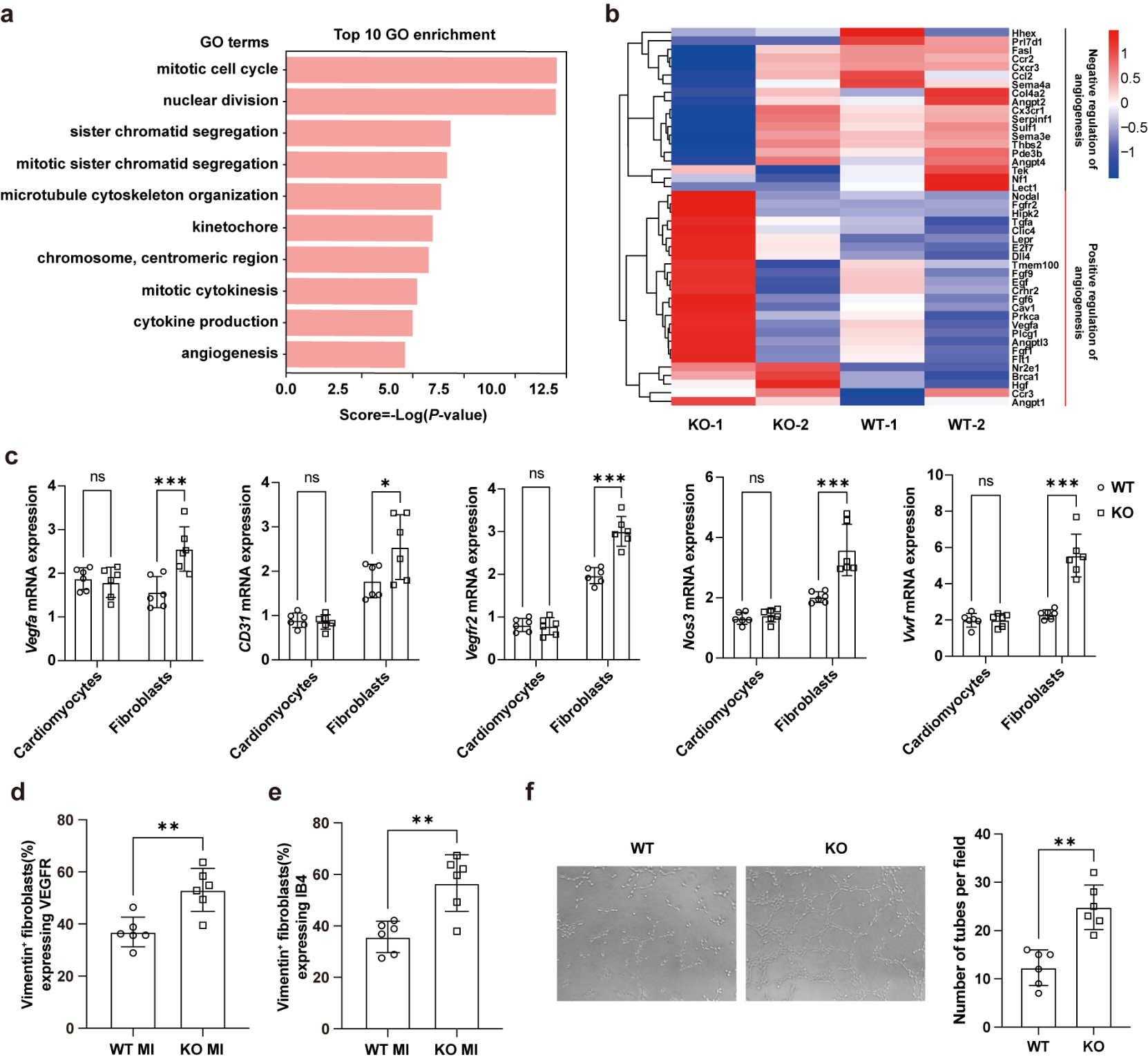


**Supplementary Fig. 7 KDM5B deficiency promotes the transformation of fibroblasts into endothelial-like cells and angiogenesis.**

**a** Top 10 Gene Ontology enrichment analysis of the differentially expressed genes in myocardial tissues from KDM5B KO and littermate control WT mice at day 7 after MI. **b** Heatmap showing differentially expressed genes relative with the positive and negative regulation of angiogenesis in myocardial tissues from KDM5B KO and WT mice at day 7 after MI operation. **c** Q-PCR analysis of *Vegfa*, *Cd31*, *Vegfr2*, *Nos3* and *Vwf* mRNA expression in cardiac fibroblasts and cardiomyocytes isolated from KDM5B KO or littermate control WT mice at day 7 after MI (n = 6 mice per group). **d**, **e** Percentage of Vimentin-positive cells expressing the endothelial markers VEGFR and IB4 through immunofluorescence staining in myocardial tissues from KDM5B KO or littermate control WT mice at day 14 post-MI (n = 6 mice per group; relative to Fig. 6e, f). **f** Representative images showing tube formation of fibroblasts isolated from KDM5B KO or littermate control WT mice. **p* < 0.05, ***p* < 0.01, ****p* < 0.001, Unpaired Student's t-test (c, d, e, f) was performed.


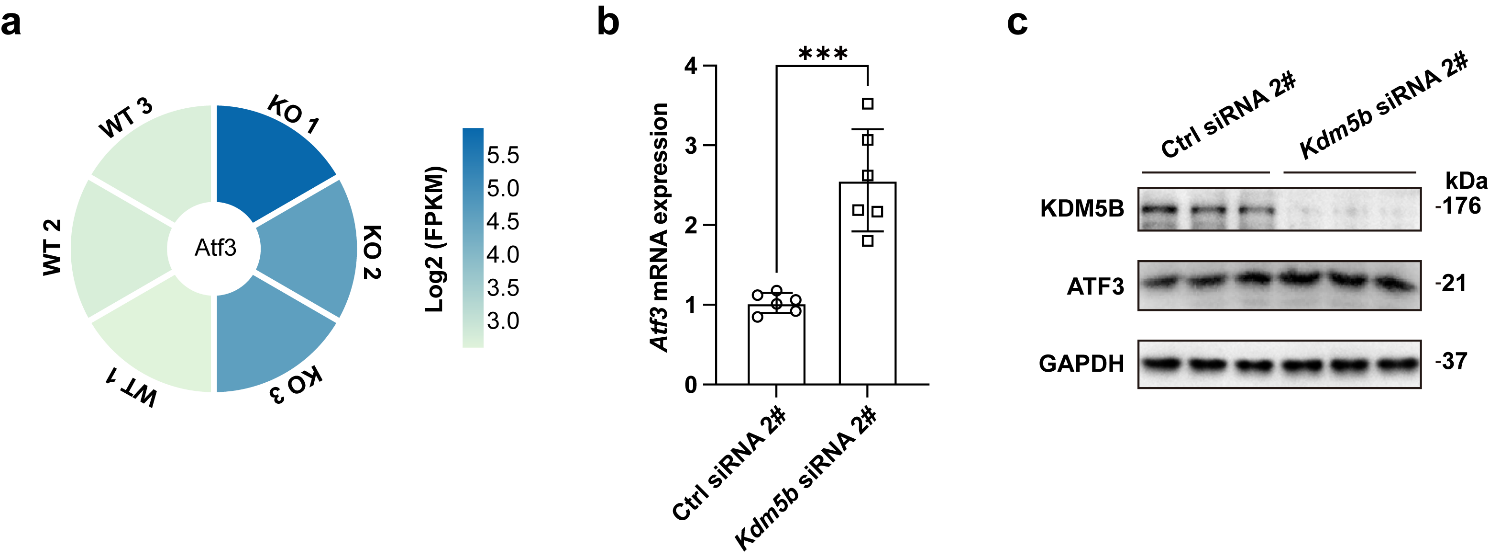


**Supplementary Fig. 8 KDM5B deficiency promotes the expression of ATF3 in cardiac fibroblasts.**

**a** Heatmap showing the upregulated expression of *Atf3* in cardiac fibroblasts isolated from KDM5B KO or littermate control WT mice at day 7 after MI. **b**, **c** Q-PCR analysis of *Atf3* mRNA expression (b) (n = 6 per group) or immunoblot analysis of ATF3 protein expression (c) in *Kdm5b*-silenced or control siRNA-transfected cardiac fibroblasts stimulated with TGF-β (10 ng/ml) for 24 h. ****p* < 0.001. Unpaired Student's t-test (b) was performed.


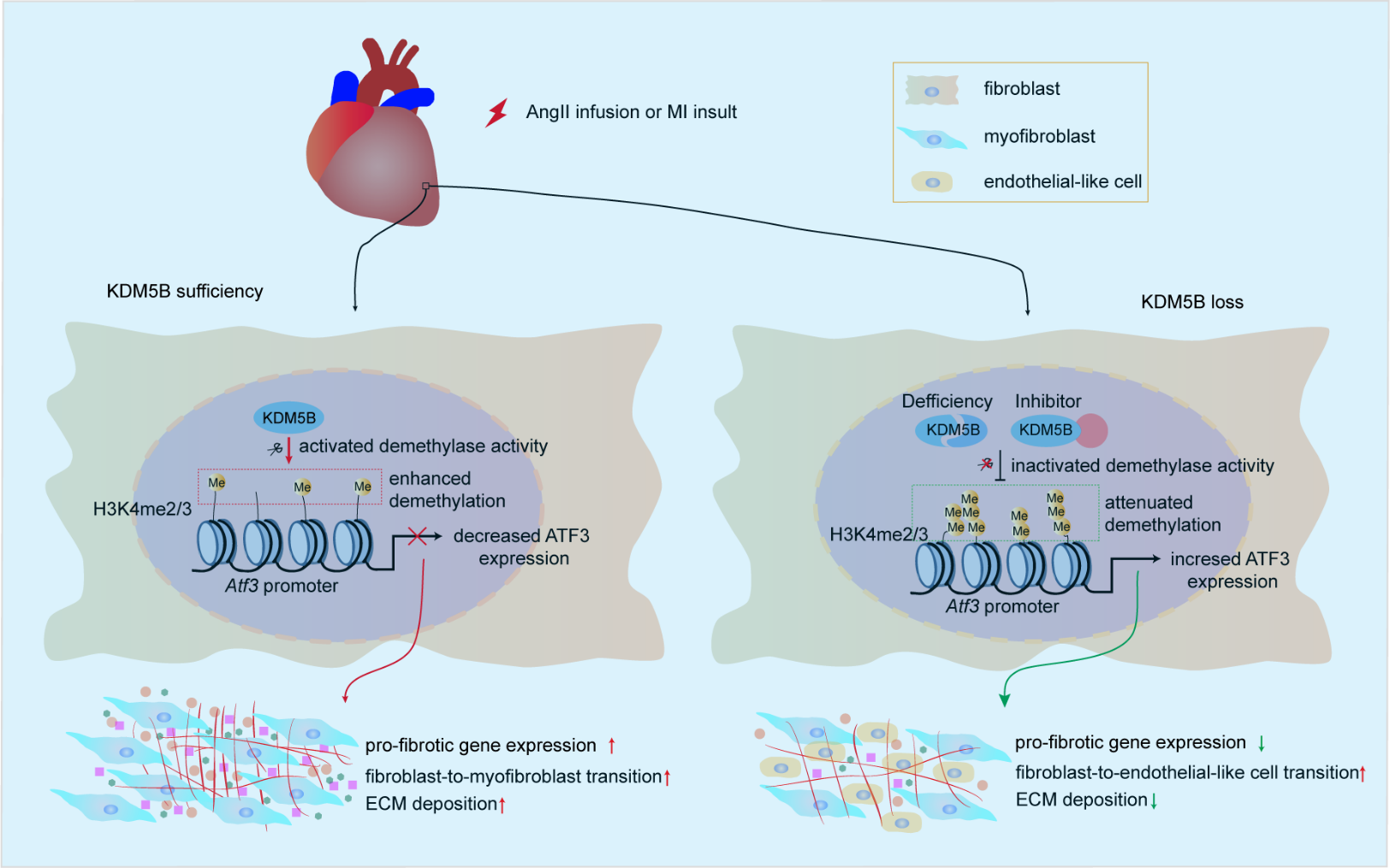


**Supplementary Fig. 9. Schematic diagram of KDM5B loss in preventing the pathological** **cardiac fibrosis.**

The epigenetic modifier KDM5B directly demethylates H3K4me2/3 of *Atf3* promoter and inhibits ATF3 expression, leading to the excessive cardiac fibrosis and cardiac dysfunction following ischemic and hypertrophic injury. KDM5B deficiency or pharmacological inhibition can decrease ECM deposition, promote angiogenesis mediated by fibroblast to endothelial-like cell transition and ameliorate pathological cardiac fibrosis.
